# A Patient Infected with SARS-CoV-2 Presenting with Complete Heart Block

**DOI:** 10.1155/2021/5011294

**Published:** 2021-08-05

**Authors:** Bindu Gyawali, Bikash Baral, Sangam Shah, Sutap Yadav, Chandra Mani Poudel

**Affiliations:** ^1^Maharajgunj Medical Campus, Institute of Medicine, Tribhuvan University, Maharajgunj, 44600, Nepal; ^2^Department of Cardiology, Manmohan Cardiothoracic Vascular and Transplant Center, Nepal; ^3^Maharajgunj Medical Campus, Institute of Medicine, Tribhuvan University, Maharajgunj 44600, Nepal

## Abstract

Complete heart block is a rare presentation in a patient with COVID-19 infection that may result when the virus enters the myocardial cell by the angiotensin-converting enzyme-2 receptor. Here, we report a case of forty-nine-year male with COVID-19 with complete heart block (CHB).

## 1. Introduction

COVID-19 is a serious illness caused by severe acute respiratory syndrome coronavirus-2 (SARS-CoV-2) which has symptoms that range from asymptomatic to moderate respiratory symptoms and can develop to potentially fatal cardiovascular and pulmonary problems. Cardiac complications include acute myocardial infarction (MI), arrhythmias, heart block, and cardiomyopathy [1]. Although respiratory problems have been widely documented in coronavirus infection, evidences suggest that cardiac involvement may result in poor outcomes [2]. The exact mechanism of this poor outcome is not understood; however, it is believed that the virus enters cardiac and vascular cells through the angiotensin-converting enzyme-2 (ACE-2) receptor. This increases the risk of direct injury to the conduction system, which might result in heart block [3]. We report a case of a forty-nine-year male with COVID-19 with complete heart block (CHB) and COVID-19.

## 2. Case Presentation

A forty-nine-year male with a history of hypertension was admitted to our hospital with chief complaints of fatigue, palpitation, uneasiness, and chest heaviness on exertion for nine days. He had no history of syncope, edema, and shortness of breath, fever, and cold intolerance. He was an occasional smoker and used to consume alcohol. He was suspected with the provisional diagnosis of suspected COVID-19 infection. He had no history of taking herbal or over-the-counter medications.

He was conscious, cooperative, and well oriented to time, place, and person at the time of presentation. On examination, his blood pressure was 100/60 mm of Hg, pulse rate was 48 beats/min which was regular with low volume, respiratory rate was 14 breaths/min, oxygen saturation was 96% in room air, and axillary temperature was 36.5°C. Systemic examination was unremarkable.

**Figure 1:**
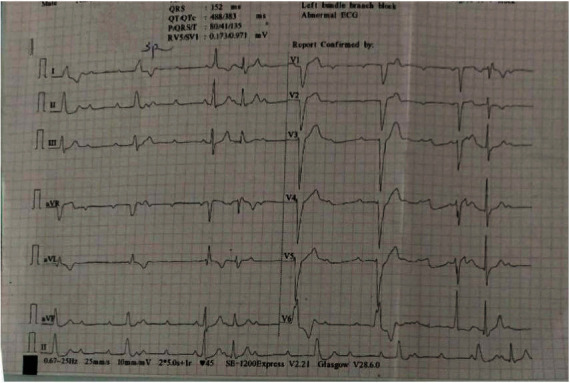
ECG of the patient done at the time of admission. ECG showed a wide QRS complex with no relation between the P wave and QRS complex.

His laboratory investigations revealed hemoglobin 11.9 gram % and packed cell volume 35.9%. He had an elevated level of alanine aminotransferase (ALT) (86 units per liter). His renal function test and thyroid function test were normal. Cardiac markers were within the normal range (creatinine kinase-MB (17 units per liter) and Troponin I (<0.012 nanograms per milliliter)). C-reactive protein (CRP) was positive (++) and Erythrocyte Sedimentation Rate (ESR) was 20/1^st^ hour. On serological examination, Hepatitis B Surface Antigen (HBsAg) immunochromatography was nonreactive. COVID-19 was confirmed positive by Reverse Transcriptase Polymerase Chain Reaction (RT-PCR). His ECG showed a wide QRS complex with no relation between the successive P wave and the QRS complex (Figure 1). The rate of the P wave was 100 beats/min, and the rate of the QRS complex was 38 beats/min which was suggestive of complete heart block.

**Figure 2:**
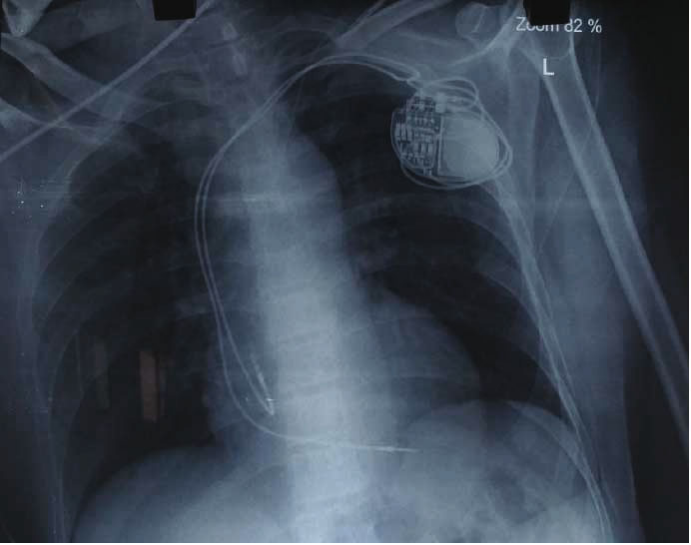
Chest X-ray showing dual-chamber permanent pacemaker (pulse generator with leads in right atrium and right ventricle).

**Figure 3:**
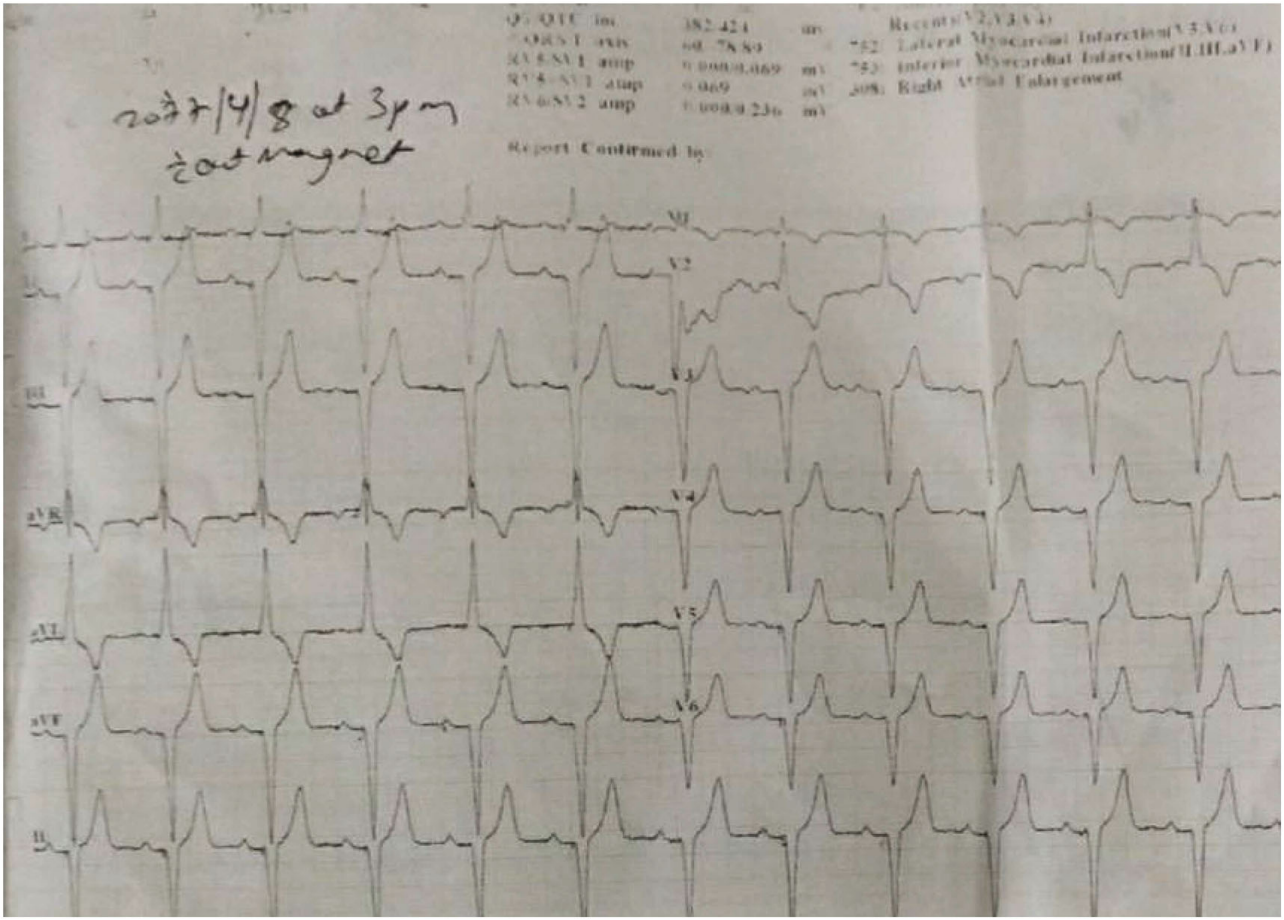
ECG of patient without magnet done after pacemaker insertion showing AsVP (atrial-sensed and ventricular-paced beats).

**Figure 4:**
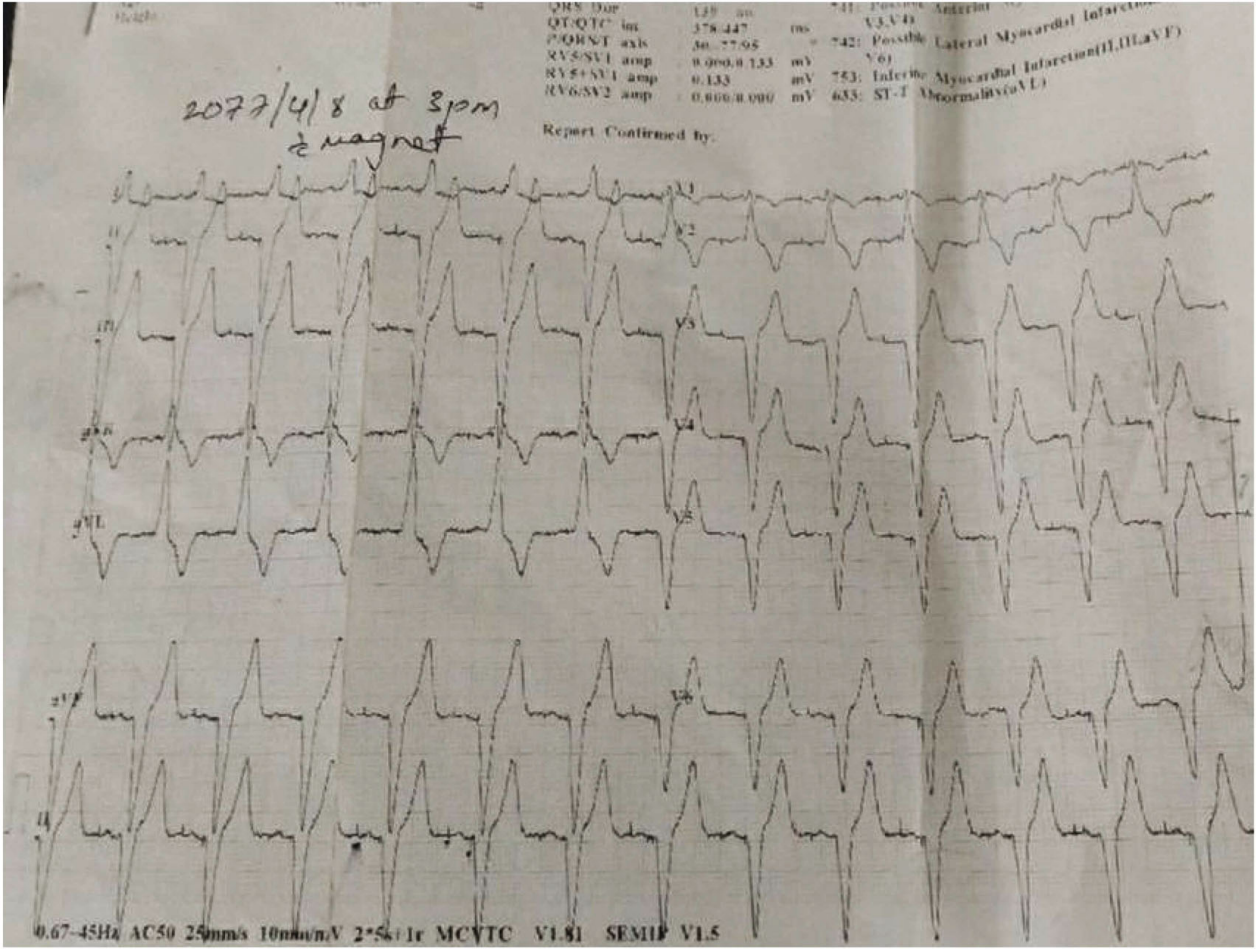
ECG of the patient with magnet after pacemaker implantation showing ApVp (both atrial- and ventricular-paced beats).

Diagnosis of third-degree complete heart block with COVID-19 was made. His echocardiogram findings were normal with a left ventricle ejection fraction of 60%. The patient was admitted to the isolation ward for the symptomatic medical management. A repeat COVID-19 RT-PCR test after 10 and 12 days of admission was confirmed negative. Dual chamber permanent pacemaker implantation was done after 14 days without procedural complications (Figure 2). After insertion of a permanent pacemaker, there was a significant improvement in his symptoms (Figures 3 and 4), and following this, he was discharged from the hospital on oral medications.

## 3. Discussion

COVID-19 infection's ability to induce cardiac damage has been the subject of considerable discussion. Hypoxemia, electrolyte abnormalities, microthrombi, an inflammatory surge from the cytokine storm, and direct invasion of the cardiomyocytes are some of the probable causes causing cardiac injury [4]. Inflammatory cells and the SARS-CoV-2 virus, if found in autopsy results, imply direct cardiac invasion. A fifty-year male patient with COVID-19 showed interstitial mononuclear inflammatory cells suggesting an inflammatory process typical of myocarditis in one of the case reports [5].

Ischemic heart disease, thyroid dysfunction, drugs, and infections are thought to be the cause of the new-onset AV node disease [6]. In our case, there was no derangement of the cardiac marker or thyroid markers; no medications before hospitalization were given that ruled out these etiologies. Thus, either focal involvement or secondary cause of AV block causing critical illness could be the probable cause in our patient. Besides, injury to the conduction system can be related to direct virus invasion. High-degree heart block caused by an infectious organism is rare. Complete heart block caused by a virus is noted in few studies. SARS and MERS have no reported cases of atrioventricular block among infected patients [7]. In this case, COVID-19 may have induced subclinical myocarditis, which resulted in high-degree AV block; this is the most likely mechanism. ACE-2 receptors are present in macrophages, endothelial cells, smooth muscle cells, and cardiomyocytes [8]. In animal models, ACE-2 receptors were found in sinoatrial nodal cells in rats, and their overexpression caused conduction problems and ventricular fibrillation [9, 10]. Another potential is SARS-CoV-2 infection of the AV node and infra-Hisian conduction pathway, resulting in AV block. Some studies suggest the role of hydroxychloroquine used in the treatment of COVID-19 as the probable cause of AV block. Hydroxychloroquine can lead to prolongation of QT intervals, and a notable side effect is the fascicular block which consequently results in an atrioventricular block.

We believe the patient was originally infected with the coronavirus, and the systemic inflammatory response to multiple organ systems may have damaged the heart's conduction system, resulting in total heart block. Complete heart block in this patient may just be an incidental finding without symptom in a patient with COVID-19 infection.

## 4. Conclusion

COVID-19's spectrum is broadening. SARS-CoV-2 viral RNA has been discovered in the heart, raising the possible risk of direct harm to the cardiac conduction system and cardiac myocytes. This may form a basis for the manifestation of complete heart block in the COVID-19 patient with cardiac involvement. However, it should be emphasized that CHB may be a rare consequence of the coronavirus infection. Furthermore, the link between CHB and coronavirus should be explored.

## Data Availability

All the available data are available in the manuscript itself.
